# Prognostic Implications of Diabetes Insipidus in Heart Failure Hospitalizations: Insights from the U.S. National Readmissions Database 2016–2021

**DOI:** 10.3390/jcm14072308

**Published:** 2025-03-28

**Authors:** Lakshmi Menon, Shubhadarshini Pawar, Dileep Kumar Reddy Regalla

**Affiliations:** 1 Division of Endocrinology and Metabolism, Department of Internal Medicine, University of Arkansas for Medical Sciences, Little Rock, AR 72205, USA; 2 Department of Interventional Cardiology, Smidt Heart Institute, Cedars Sinai Medical Center, Los Angeles, CA 90048, USA; shubha.pawar9@gmail.com; 3 Department of Hospital Medicine, OSF Saint Anthony Medical Center, Rockford, IL 61107, USA; dileepkumar.r.regalla@osfhealth.org

**Keywords:** diabetes insipidus, arginine vasopressin deficiency, arginine vasopressin resistance, heart failure, readmission

## Abstract

**Background:** Diabetes insipidus affects heart failure outcomes through its impact on volume status and electrolyte imbalance. However, previous data on its impact on heart failure hospitalizations are limited. This study aimed to evaluate the prognostic implications of diabetes insipidus in patients admitted with heart failure. We utilized the United States National Readmissions Database (NRD) from the years 2016 to 2021. **Methods:** Adult patients hospitalized with a primary diagnosis of heart failure were stratified based on the presence of diabetes insipidus. Propensity matching was used to balance the baseline characteristics. Multivariable logistic regression was used to estimate the association of heart failure with diabetes insipidus on clinical outcomes, complications, 30-day readmissions, and healthcare utilization. **Results:** Among 5,946,749 heart failure hospitalizations, 2846 (0.04%) had a secondary diagnosis of diabetes insipidus. Compared with matched control, patients with heart failure and diabetes insipidus had significantly higher in-hospital mortality (odds ratio [OR] 5.77 [95% CI, 4.78–6.97], *p* < 0.001). Patients with heart failure and diabetes insipidus were also associated with increased odds of acute kidney injury (OR 2.11 [95% CI, 1.86–2.39], *p* < 0.001), hypernatremia (OR 4.98 [95% CI, 1.86–2.39], *p* < 0.001), cardiogenic shock (OR 1.69 [95% CI, 1.32–2.15], *p* < 0.001), and cerebral edema (OR 22.28 [95% CI, 14.74–33.69], *p* < 0.001) compared with the matched controls. No difference was found in the all-cause readmission (OR 0.89 [95% CI, 0.76–1.04], *p* = 0.14), but patients with diabetes insipidus had a lower risk of heart failure readmissions (OR 0.47 [95% CI, 0.33–0.66], *p* < 0.001) and a higher risk of non-cardiac readmissions (OR 2.21 [95% CI, 1.48–3.9], *p* < 0.001). **Conclusions:** Diabetes insipidus was associated with worse outcomes in patients with primary heart failure hospitalizations, which was likely secondary to the risk of excessive diuresis.

## 1. Introduction

Diabetes insipidus is characterized by the excretion of a large volume of hypotonic urine due to the impaired production or action of the hormone arginine vasopressin (AVP). The urine output exceeds 50 mL per kg body weight per 24 h, leading to increased thirst and water intake [1]. If the fluid loss is not compensated for with increased water intake, the patients develop hypernatremia and volume depletion.

AVP is synthesized in the magnocellular neurons of the paraventricular and supraoptic nuclei of the hypothalamus and transported via the supraoptic hypophyseal tract to be stored in secretory granules in the posterior pituitary [2]. The primary trigger for AVP release is an increase in plasma osmolarity. Other factors that stimulate AVP release are a reduced circulating volume, nausea, vomiting, stress, hypoxia, and exercise [3]. AVP secretion is inhibited by reduced plasma osmolarity, increased plasma volume, alcohol consumption, and opioids [2]. Diabetes insipidus is classified into central diabetes insipidus when the defect involves impaired production and the secretion of AVP or nephrogenic diabetes insipidus when the kidneys are resistant to the action of AVP [4]. Additionally, gestational diabetes insipidus in an entity is seen during pregnancy when there is increased metabolism of AVP due to the production of placental vasopressinase, which leads to decreased circulating levels of AVP [5]. The prevalence of diabetes insipidus is approximately 1 per 25,000 in the general population [1]. The Working Group for Renaming Diabetes Insipidus has recommended changing the name of central diabetes insipidus to AVP deficiency and nephrogenic diabetes insipidus to AVP resistance, respectively [6]. The rationale for this name change is to avoid confusing the disease with diabetes mellitus, which may lead to inappropriate blood glucose measurements, the prescription of diabetes medications, and the withholding of desmopressin for the treatment of diabetes insipidus. Since the International Classification of Diseases, Tenth Revision (ICD-10) uses the term diabetes insipidus, we use this term in our manuscript.

Heart failure is a growing public health challenge, with approximately 6.7 million Americans affected by it [7]. Nearly one in four patients are readmitted within 30 days of discharge for a heart failure exacerbation [8]. One of the primary goals of treatment during a heart failure hospitalization is to relieve the volume overload and congestion [9]. Diabetes insipidus can impact heart failure through its effects on volume status and urine output. Additionally, the medications used for the treatment of diabetes insipidus to reduce urine output can also have an impact on heart failure by promoting fluid retention [1]. To our knowledge, no previous study has evaluated the impact of diabetes insipidus on heart failure hospitalizations on a population basis. Such data would provide insight into the burden of DI in patients admitted with heart failure. Understanding the impact of diabetes insipidus on heart failure hospitalizations can also help hospitals and physicians develop better strategies to manage this patient population.

The primary objective of this study was to determine the effect of diabetes insipidus on all-cause mortality among primary hospitalizations for heart failure using the National Readmission Database (NRD). The secondary objectives were to evaluate how diabetes insipidus affects the risk of various co-morbidities, including acute kidney injury and hyponatremia. We also evaluated the rate of readmissions and the impacts on cost and length of stay in our study population.

## 2. Methodology

The National Readmissions Database, a component of the Healthcare Cost and Utilization Project funded by the Agency for Healthcare Research and Quality, offers extensive data on hospital admissions across diverse healthcare facilities in the United States [10]. This dataset encompasses approximately 60% of all hospitalizations in the United States and is derived from 84% of discharges recorded in State Inpatient Databases, without limitations regarding payer status. This analysis included data from 1 January 2016 to 31 December 2021. Unweighted, it contains data from approximately 7 million hospital stays each year. When weighted, it estimates around 35 million hospitalizations nationally [10]. The dataset monitors readmissions by utilizing patient linkage information to identify the same individual across hospitals within a state over a calendar year. Approval from the Institutional Review Board and informed consent were not required because the dataset is publicly accessible and de-identified.

### 2.1. Study Population

**Figure 1:**
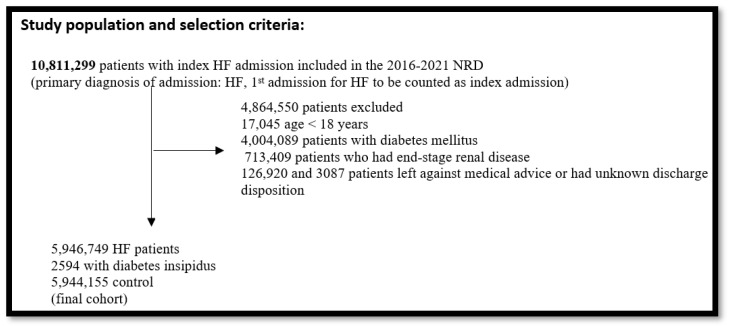
Study data selection flowchart: This yielded a final study population of 5,946,749 heart failure patients. We further stratified the heart failure patients into patients with diabetes insipidus and without diabetes insipidus (Table 1).

We included adult (≥18 years) patients with index heart failure hospitalization in the calendar year using the International Classification of Diseases, Tenth Revision, and Clinical Modification (ICD 10-CM) codes (Supplementary Section). Index hospitalization was defined as the first hospitalization in the calendar year for heart failure. Since the NRD does not allow for year-to-year linkage, the patients and hospitals from each year were considered separate entities. The ICD codes for heart failure have high positive predictive value (>90%) and specificity (>95%) but low sensitivity (>50%) [11,12]. We excluded patients who had left against medical advice, who had unknown discharge disposition, in the pediatric population, and who had end-stage renal disease and type 2 diabetes mellitus (Figure 1).

The baseline patient demographics (age, sex, median household income, primary expected payer); calendar year of admission; comorbidities, such as smoking, dyslipidemia, hypertension, obesity, anemia, diabetes, heart failure, known coronary artery disease, prior myocardial infarction, prior coronary artery bypass grafting, chronic kidney disease, cancer, and those included in the Elixhauser Comorbidity Index; and hospital characteristics, such as bed size, urban vs. rural location, and teaching vs. non-teaching status were extracted from the database. ICD-10-CM, ICD-10-PCS (Procedure Classification System), and Elixhauser comorbidity software were used to identify the comorbidities and patient characteristics (Supplementary Table S1).

### 2.2. Outcomes

The primary outcome of this study was in-hospital mortality. Secondary outcomes of this study included total hospitalization costs, length of stay, acute kidney injury, hyponatremia, hypernatremia, acute pulmonary edema, chronic pulmonary edema, cerebral edema, urinary tract infections (UTIs), vasopressor use, mechanical ventilation, cardiogenic shock, septic shock, ventricular tachycardia, and 30-day all-cause and cause-specific (cardiac, non-cardiac, heart failure) readmissions. The Refined Clinical Classifications Software (CCSR) for ICD-10-CM Diagnoses established for the Health Care Utilization Project were employed to identify cause-specific readmissions (CIR001-CIR039 for cardiac conditions, CIR019 for heart failure, and the remainder classified as non-cardiac) [13].

### 2.3. Statistical Analysis

Statistical analysis was performed using Stata, version 18 (Statistical Software: Release 18. College Station, TX: Stata Corp LLC). Categorical variables were presented as the frequency (percentage) and continuous variables as the median (interquartile range). Odds ratios (ORs) and 95% confidence intervals (95% CIs) were used to report the result of the propensity-weighted analysis. A 1:1 propensity score matching was performed to account for the non-random treatment assignment (with diabetes insipidus vs. no diabetes insipidus). Propensity scores were estimated using a non-parsimonious multivariable logistic regression model that was adjusted for the following covariates—age; sex; median household income; primary expected payer; calendar year of admission; comorbidities, such as smoking, dyslipidemia, hypertension, obesity, anemia, diabetes, heart failure, known coronary artery disease, prior MI, prior coronary artery bypass grafting, chronic kidney disease, cancer, and those included in the Elixhauser Comorbidity Index; and hospital characteristics, such as bed size, urban vs. rural location, and teaching vs. non-teaching status from Table 1. The variables incorporated into the multivariable logistic regression model were chosen based on both statistical and clinical significance. Initially, the variables that exhibited a *p*-value < 0.20 in the univariable analysis were deemed eligible for inclusion. Furthermore, the clinically significant factors recognized to affect heart failure outcomes (e.g., age, sex, comorbidities, and hospital features) were included in the model irrespective of their statistical significance. We computed the Variance Inflation Factor (VIF) for all independent variables to evaluate the multicollinearity. No variables surpassed the widely accepted VIF threshold of 10, signifying a lack of significant collinearity. The selection of models was enhanced through the application of the Akaike Information Criterion (AIC) and Bayesian Information Criterion (BIC) to achieve an optimal equilibrium between the model fit and complexity. Considering these factors, we recognize that variable selection may have led to potential residual confounding, representing a limitation of our study.

Secondary outcomes, including the cost and length of stay, were evaluated using Poisson regression. Our post hoc analysis showed a power of 1.0 at a significance level of 0.05. This indicates that this study had sufficient statistical power to detect meaningful differences. We also performed multivariable logistic regression models for 30-day readmissions.

In accordance with previous research on hospital-level estimations, unweighted data were utilized for the analysis [13,14]. The weighted estimates in the NRD pertain to discharge-level data and do not apply to hospital-level studies following the 2012 database redesign [15]. The dataset’s complex survey design was addressed using clustering and strata variables, as advised in the Agency for Healthcare Research and Quality Methods Series [16]. Missing data were addressed using simple imputation, utilizing the predominant category for categorical variables and the median value for continuous variables. The incidence of missing data was minimal: 0.13% for payer status, 1.32% for median household income quartile, 0.4% for hospital location, and 0.04% for mortality status. All *p*-values were two-sided with a significance threshold of <0.05. This study followed the Strengthening the Reporting of Observational Studies in Epidemiology (STROBE) reporting guidelines.

## 3. Results

### 3.1. Demographics and Baseline Comorbidities

A total of 10,811,299 patients were found in the 2016 to 2021 national readmission database (NRD) with heart failure as the primary diagnosis of admission. After applying the exclusionary criteria, 5,946,749 patients were included in this study. Among these, 2594 patients had underlying diabetes insipidus. The baseline characteristics of heart failure hospitalizations with and without co-existing diabetes insipidus are shown in Table 1. The patients with co-existing diabetes insipidus were younger (64 vs. 72 years, *p* < 0.001); however, this difference was not significant after the propensity score matching (PSM). All significant imbalances in the baseline covariates before matching were balanced after matching. Multivariate regression analysis did not reveal substantial differences in the odds of underlying comorbidities, as listed in the table. The patients had a median age of 64, and 48% were women. The majority of patients had Medicare insurance (60%). In addition, the majority of patients were treated at large hospitals (64%) in an urban location (86%). The most common comorbidities in both diabetes insipidus and non-diabetes insipidus cohorts included dyslipidemia (35.4% vs. 34.6%), hypothyroidism (28.2% vs. 27.8%), chronic lung disease (27.4% vs. 26.7%), and chronic kidney disease (25.7% vs. 24.7%). The baseline characteristics and comorbidity burden of diabetes insipidus and non-diabetes insipidus cohorts were not statistically significant except for prior MI (6.8% vs. 5.3%, *p* = 0.03); however, this was a minor variance (Table 1).

### 3.2. In-Hospital Outcomes

The patients with both heart failure and diabetes insipidus had a higher in-hospital mortality (OR 5.77, 95% CI: 4.78–6.97, *p* < 0.001). The median costs of hospitalization in the diabetes insipidus group and non-diabetes insipidus group were USD 25,299 and USD 12,898, respectively (USD 12,401/55% higher in the diabetes insipidus group; adjusted coefficient: 0.55, 95% CI: 0.54–0.56, *p* < 0.001). The diabetes insipidus group also had a median length of stay of 7 days compared with 5 days for the non-diabetes insipidus group (adjusted coefficient: 0.44, 95% CI: 0.42–0.45, *p* < 0.001).

### 3.3. In-Hospital Complications

The patients in the diabetes insipidus cohort were found to have higher odds of developing acute kidney injury (AKI) (OR 2.11, 95% CI: 1.86–2.39, *p* < 0.001), hypernatremia (OR 4.98, 95% CI: 4.08–6.08, *p* < 0.001), cerebral edema (OR 22.28, 95% CI: 14.74–33.69, *p* < 0.001), and UTIs (OR 1.59, 95% CI: 1.35–1.87, *p* < 0.001). The patients in the diabetes insipidus cohort were also more likely to require mechanical ventilation (OR 5.23, 95% CI: 4.46–6.14, *p* < 0.001) and vasopressor support (OR 3.07, 95% CI: 2.35–3.99, *p* < 0.001) (Table 2). In addition, the patients in the diabetes insipidus cohort had higher odds of developing septic shock (OR 1.69, 95% CI: 1.32–2.15, *p* < 0.001) and cardiogenic shock (OR 2.86, 95% CI: 2.34–3.49, *p* < 0.001).

In contrast, the patients in the diabetes insipidus cohort had lower risks of hyponatremia (OR 0.77, 95% CI: 0.66–0.91, *p* < 0.001) and atrial fibrillation (OR 0.61, 95% CI: 0.53–0.69) compared with the non-diabetes insipidus cohort. However, the NRD dataset did not distinguish between preexisting atrial fibrillation and new onset atrial fibrillation during the hospitalization. There were no differences in the odds of developing acute or chronic pulmonary edema between the two groups (Table 2).

### 3.4. Thirty-Day Readmissions

The patients in the diabetes insipidus cohort had lower odds of heart failure readmissions (216 readmissions vs. 293 readmissions in the non-diabetes insipidus cohort) (OR 0.47, 95% CI: 0.33–0.66, *p* < 0.001). Similarly, the patients in the diabetes insipidus group had lower odds of cardiac readmissions (265 readmissions vs. 333 readmissions in the non-diabetes insipidus group) (OR 0.45, 95% CI: 0.30–0.67, *p* < 0.001). In contrast, the diabetes insipidus cohort has higher odds of readmission from non-cardiac causes (93 readmissions vs. 60 readmissions in the non-DI cohort) (OR 2.21, 95% CI: 1.48–3.29, *p* < 0.001). In addition, no significant difference in the all-cause readmissions was found between the two groups (Table 3).

## 4. Discussion

This retrospective analysis of the NRD compared the outcomes of hospitalization for heart failure in patients with diabetes insipidus with those without diabetes insipidus. No significant variation existed in the baseline characteristics and comorbidity burden between the diabetes insipidus and non-diabetes insipidus groups after propensity matching, except for one small variance in the history of prior MI. This study’s key findings were that compared with heart failure hospitalizations without diabetes insipidus, those with a diagnosis of diabetes insipidus during the index hospitalization had (A) higher in-hospital mortality; (B) higher risks of developing AKI, UTIs, cerebral edema, hypernatremia, septic shock, and cardiogenic shock; (C) higher needs for mechanical ventilation and vasopressor use; (D) a lower risk of hyponatremia; (E) increased readmission for non-cardiac causes; (F) lower readmission for heart failure; and (G) an increased length of stay and cost of hospitalization.

Patients with central diabetes insipidus can experience more rapid diuresis when the desmopressin dose is withheld or lowered, leading to excess free water loss compared with sodium loss [17]. Similarly, in nephrogenic diabetes insipidus, withholding pharmacological treatment with thiazide diuretics or amiloride leads to an increase in the urine output [18]. This rapid diuresis can also account for the increased rates of hypernatremia that we found in this population. Atrial fibrillation is linked to worsening pulmonary congestion. The low odds of atrial fibrillation in our study could potentially contribute to rapid diuresis. The disadvantage of rapid diuresis is that it can lead to severe dehydration and decreased intravascular volume. Indeed, hypernatremia is predictive of an increased risk of mortality and readmission at 12 months in patients with heart failure, regardless of the left ventricular injection fraction [19]. Our study did find increased rates of acute kidney injury and an increased need for vasopressor support among the patients with diabetes insipidus, both of which are explained by more rapid diuresis. Other factors that can lead to an increased risk of hypernatremia are altered mental status, sedation in the ICU, and physical weakness affecting the ability to drink. We theorize that the rapid correction of hypernatremia was responsible for the increased rate of cerebral edema seen in the patients with diabetes insipidus [20]. The increased odds of mechanical ventilation in the diabetes insipidus group are explained by the increased odds of cardiogenic shock and the increased risk of neurological dysfunction and respiratory failure secondary to hypernatremia and cerebral edema.

Hyponatremia is a common finding at admission for heart failure, with a reported prevalence of 20–29% [21,22]. Hyponatremia in heart failure is typically dilutional and reflects the volume overload. AVP plays a role in the hyponatremia and volume overload seen in congestive heart failure. AVP levels are elevated in patients with both acute and chronic heart failure, where it was shown to cause vasoconstriction, left ventricular remodeling, and water retention [23]. Several studies looked into the prognostic implication of hyponatremia in patients with heart failure and found that it is associated with increased mortality [22,24]. The patients with diabetes insipidus admitted for heart failure had less hyponatremia compared with those without diabetes insipidus. The reduced rates of hyponatremia seen in the patients with diabetes insipidus can be secondary to a decreased volume overload due to the impaired release or action of AVP.

Patients with diabetes insipidus have an increased heart rate, left ventricular contractility, and impaired diastolic function, even though they maintain a similar blood pressure compared with controls [25]. Desmopressin administration leads to a reduction in the heart rate and normalization of the echocardiographic parameters. Thus, withholding desmopressin has adverse effects on cardiac function. This can account for the increased rates of cardiogenic shock seen in patients with diabetes insipidus.

The patients with diabetes insipidus had a lower risk of cardiac readmission. An explanation for this finding is that this patient population had a lower burden of cardiovascular disease at the baseline. In Table 1, an increased prevalence of prior MI was noted in the control population without diabetes insipidus. Additionally, due to the higher in-hospital mortality in the diabetes insipidus group, fewer patients were available for readmission. This introduced survival bias, as the patients who survived were not necessarily representative of the entire initial patient group. This may have led to an underestimation of the true readmission risk in patients with diabetes insipidus. The increase in non-cardiac hospitalization in patients with diabetes insipidus could be secondary to the increased prevalence of co-morbidities, such as adrenal insufficiency. Adrenal insufficiency is associated with an increased risk of metabolic co-morbidities, such as diabetes, as well as an increased risk for infection [26]. Patients with adrenal insufficiency also have an increased risk of all-cause hospitalization, increased length of stay, and 1-year readmissions compared with controls [26]. In contrast, a study that looked at the risk of mortality among hospitalized patients with hypopituitarism showed that patients with diabetes insipidus had a 3-fold increased risk of in-hospital death, but no increased mortality was seen in patients with hypopituitarism without diabetes insipidus [27].

Hospital characteristics may contribute to the difference in outcomes seen between the two groups. The patients with diabetes insipidus were more likely to be admitted to teaching hospitals. PSM was performed to balance this variable but did not completely eliminate the imbalance. Teaching hospitals have more access to advanced technology, specialists, and multidisciplinary teams, which can aid in the management of complicated conditions. However, the involvement of multiple care teams can lead to more extensive testing and an increased cost of hospitalization and length of stay. Teaching hospitals are more likely to serve patients with higher acuity, which contributes to the increased healthcare costs [28]. Ultimately, teaching hospitals offer specialized care to more complex patients but have higher healthcare costs.

Since AVP levels are elevated in heart failure, the vasopressin receptor blockade was studied as a target for therapy. The EVEREST randomized, double-blind trials of the V2 receptor antagonist Tolvaptan in patients with reduced left ventricular ejection fraction (≤40%) hospitalized for exacerbation of heart failure showed positive effects on fluid balance and dyspnea but did not demonstrate an improvement in long-term mortality [29]. In the SECRET clinical trial, the addition of Tolvaptan to the background diuretic regimen in patients hospitalized with acute heart failure with either reduced or preserved ejection fraction did not lead to a difference in dyspnea reduction on day 1 but showed greater dyspnea reduction on day 3 [30]. Currently, the role of vasopressin antagonists in acute heart failure is limited to situations where there is persistent hyponatremia and fluid overload despite water restriction and the use of loop or thiazide diuretics. Vasopressin antagonists help relieve congestion while maintaining the serum sodium [8]. Studying patients with diabetes insipidus can provide insights into the expected changes from a combined V1a and V2 receptor blockade in patients with heart failure.

Sodium-glucose cotransporter 2 inhibitors (SGLT2 inhibitors) were shown to reduce readmissions for heart failure, slow down the progression of chronic kidney disease, and improve glycemic control [9,31]. The use of SGLT2 inhibitors in patients with heart failure is associated with an increase in the co-peptin level, a marker for AVP release, which promotes water conservation in the setting of the osmotic diuretic effect of glycosuria and prevents a significant increase in urine volume [32]. This indicates that patients with diabetes insipidus may be more prone to fluctuations in urine output in the setting of SGLT2 inhibitor use due to impaired endogenous AVP production or action. While SGLT2 inhibitors are approved for use in the ambulatory setting in patients with chronic heart failure, they were evaluated for the treatment of acute heart failure in hospitalized patients too. The EMPULSE trial showed that the initiation of Empagliflozin for acute heart failure resulted in early and effective decongestion [33]. Caution must be exerted when considering the use of SGLT2 inhibitors in hospitalized patients with diabetes insipidus and heart failure.

The current diabetes insipidus guidelines address the impact of special circumstances, such as pregnancy and alcohol consumption, on the management of diabetes insipidus [34,35]. However, there is a notable gap in information regarding the management of diabetes insipidus in patients with congestive heart failure, and no specific treatment protocols exist for this population. Given the worse prognosis observed in these patients, there is a clear need for a tailored treatment protocol for patients with diabetes insipidus and co-existing congestive heart failure.

The main strength of this study was that we looked at a national-level database that included a large population of patients with diabetes insipidus. We excluded patients with the confounding diagnosis of diabetes mellitus, which is known to impact mortality and readmission rates in patients with heart failure admissions [36,37]. We also excluded patients with ESRD since they are resistant to the polyuric effect induced by diabetes insipidus [38]. Our study had some limitations. There could be additional confounders, such as dementia, that were not accounted for, which could affect the mortality and other clinical outcomes. We used PSM, which reduced but did not eliminate the baseline differences in the characteristics between the two groups. The SMD was <0.1 for all variables, which indicates good balancing. We relied on ICD-10 codes, which could be inaccurate or incomplete in documenting diagnoses and co-morbidities. The NRD does not provide data on medications or symptoms or document the severity of electrolyte disturbances. Similarly, the severity of heart failure, based on New York Heart Association classifications and ejection fraction, was unavailable. The NRD relies on ICD-10 codes for identifying atrial fibrillation, which do not differentiate between preexisting atrial fibrillation and new-onset atrial fibrillation during hospitalization. Consequently, atrial fibrillation cannot be reliably analyzed as an in-hospital outcome in studies utilizing the NRD. As with all studies analyzing information obtained from the NRD, we could not determine the causality and could only highlight associations. Further studies are needed to determine the causal mechanisms for the increased mortality seen in patients with diabetes insipidus.

## 5. Conclusions

In the patients with diabetes insipidus and heart failure, withholding or lowering the dose of the medication used for the treatment of diabetes insipidus could lead to rapid diuresis by promoting free water loss. However, this increased the risk of multiple in-hospital complications, such as hypernatremia, cardiogenic shock, and acute kidney injury. The patients with diabetes insipidus were also more prone to developing septic shock and UTIs during their hospitalization. This led to increases in the mortality, length of stay, and cost of admission.

## Figures and Tables

**Table 1:** Baseline patient and hospital characteristics of patients with heart failure stratified by diabetes insipidus before and after propensity score matching.

Characteristic—n (%)	Unweighted			Propensity Score Matched		
	Diabetes Insipidus (n = 2594)	Without Diabetes Insipidus (n = 5,944,155)	SMD ^†^	Diabetes Insipidus (n = 2590)	Without Diabetes Insipidus (n = 2590)	SMD ^†^
**Patient Characteristics**						
Age (years)—median (IQR)	64 (52–75)	76 (65–85)	0.75	63 (49–77)	64 (52–74)	0.06
Women	1249 (48.2)	2,952,827 (49.7)	0.04	1247 (48.2)	1242 (47.9)	0.01
**Primary Expected Payer**						
Medicare	1565 (60.5)	4,477,688 (75.4)	0.22	1572 (60.7)	1536 (59.3)	0.04
Medicaid	421 (16.3)	486,114 (8.2)		419 (16.2)	495 (19.1)	
Private	466 (18)	708,044 (11.9)		466 (17.9)	436 (16.8)	
Uninsured	85 (3.3)	124,443 (2.1)		85 (3.3)	78 (3.1)	
Others	46 (1.8)	126,567 (2.1)		46 (1.8)	41 (1.6)	
**Median Household Income, Percentile**						
0–25th	665 (26)	1,627,525 (27.7)	0.07	699 (26.9)	733 (28.3)	0.02
26–50th	646 (25.3)	1,585,547 (27)		646 (24.9)	665 (25.7)	
51–75th	633 (24.8)	1,438,871 (24.5)		633 (24.4)	659 (25.4)	
76–100th	612 (23.9)	1,214,028 (20.7)		612 (23.6)	533 (20.6)	
**Years**						
2016	360 (13.8)	880,540 (14.8)	0.03	360 (13.9)	358 (13.8)	<0.01
2017	430 (16.6)	980,784 (16.5)		426 (16.5)	418 (16.1)	
2018	411 (15.8)	1,004,580 (16.9)		411 (15.8)	423 (16.3)	
2019	472 (18.2)	1064,650 (17.9)		472 (18.2)	483 (18.6)	
2020	456 (17.6)	989,323 (16.6)		456 (17.6)	457 (17.6)	
2021	465 (17.9)	1,024,278 (17.2)		465 (17.9)	451 (17.4)	
**Comorbidities**						
Smoking	535 (20.6)	1,784,689 (30.1)	0.21	535 (20.6)	553 (21.4)	0.03
Dyslipidemia	921 (35.5)	2,868,441 (48.3)	0.25	917 (35.4)	895 (34.6)	0.01
Hypertension	504 (19.4)	1,643,818 (27.6)	0.37	502 (19.4)	457 (17.6)	0.02
Obesity	561 (21.6)	1,026,002 (17.3)	0.11	561 (21.6)	569 (21.9)	0.02
Known CAD	628 (24.1)	2,441,741 (41.1)	0.36	626 (24.2)	575 (22.2)	0.07
Prior MI	175 (6.7)	728,571 (12.3)	0.17	175 (6.8)	138 (5.3)	0.03
Prior PCI	122 (4.7)	633,862 (10.6)	0.22	122 (4.7)	95 (3.7)	0.04
Prior CABG	101 (3.8)	554,588 (9.3)	0.23	101 (3.9)	87 (3.4)	0.05
Prior TIA/stroke	194 (7.5)	581,929 (9.8)	0.09	194 (7.5)	183 (7.1)	0.02
Peripheral vascular disease	281 (10.8)	1,068,557 (17.9)	0.19	279 (10.7)	249 (9.6)	0.05
Anemia	176 (6.8)	383,060 (6.4)	0.01	176 (6.8)	161 (6.2)	0.01
Chronic kidney disease	665 (25.6)	1,711,774 (28.8)	0.07	665 (25.7)	640 (24.7)	0.02
Chronic lung disease	714 (27.5)	2,189,842 (36.8)	0.20	710 (27.4)	691 (26.7)	0.06
Chronic liver disease	269 (10.4)	366,099 (6.1)	0.15	269 (10.4)	246 (9.5)	0.02
Coagulopathy	457 (17.6)	633,448 (10.6)	0.20	457 (17.6)	428 (16.5)	0.08
Hypothyroidism	730 (28.1)	1,092,122 (18.4)	0.23	730 (28.2)	719 (27.8)	0.06
Pulmonary circulation disorders	352 (13.6)	992,286 (16.7)	0.08	352 (13.6)	351 (13.5)	0.05
Cancer	78 (3.1)	164,677 (2.8)	<0.01	78 (3.1)	99 (3.8)	<0.01
**No. of Elixhauser** **comorbidities—median (IQR)**	5 (4–7)	6 (5–7)	0.16	6 (5–7)	6 (5–7)	0.04
**Hospital Characteristics**						
**Bed Size**						
Small	362 (13.9)	1,049,228 (17.6)	0.16	362 (13.9)	320 (12.4)	0.03
Medium	631 (24.3)	1,682,046 (28.3)		631 (24.4)	607 (23.4)	
Large	1601 (61.7)	3,212,881 (54.1)		1597 (61.7)	1663 (64.2)	
**Location**						
Urban	2225 (86.1)	4,945,427 (83.5)	0.12	2229 (86.1)	2218 (85.6)	0.06
Rural	361 (13.9)	974,666 (16.5)		361 (13.9)	372 (14.4)	
**Teaching Status**						
Nonteaching	443 (17.1)	1,348,724 (22.7)	0.03	443 (17.1)	501 (19.3)	<0.01
Teaching	2021 (77.9)	4,070,850 (68.5)		2017 (77.8)	1916 (73.9)	

Represented as number (percentage) or median (interquartile range). ^†^ Standardized mean differences of 0.2 to 0.5, 0.5 to 0.8, and >0.8 represent small, medium, and large effect sizes, respectively. Abbreviations: CABG: coronary artery bypass graft; CAD: coronary artery disease; PCI: percutaneous coronary intervention.

**Table 2:** Association between diabetes insipidus and in-hospital outcomes in heart failure patients using multivariate regression model after propensity score matching.

Outcomes	Diabetes Insipidus (n = 2590)	Without Diabetes Insipidus (n = 2590)	Odds Ratio (95% Confidence Interval)		*p*-Value
			Unadjusted	Adjusted	
**In-hospital Outcomes**					
**Mortality**	683 (26.4)	176 (6.8)	4.91 (4.11–5.85)	5.77 (4.78–6.97)	<0.001
**Acute kidney injury**	1274 (49.2)	874 (33.7)	1.90 (1.69–2.12)	2.11 (1.86–2.39)	<0.001
**Hyponatremia**	342 (13.2)	417 (16.1)	0.79 (0.67–0.92)	0.77 (0.66–0.91)	<0.001
**Hypernatremia**	557 (21.5)	153 (5.9)	4.36 (3.61–5.26)	4.98 (4.08–6.08)	<0.001
**Acute pulmonary edema**	18 (0.7)	16 (0.6)	1.12 (0.57–2.21)	1.09 (0.55–2.17)	0.79
**Chronic pulmonary edema**	24 (0.93)	20 (0.77)	1.20 (0.66–2.18)	1.19 (0.65–2.20)	0.55
**Cerebral edema**	393 (15.2)	26 (1)	17.64 (11.81–26.34)	22.28 (14.74–33.69)	<0.001
**Atrial fibrillation**	719 (27.8)	949 (36.6)	0.66 (0.59–0.74)	0.61 (0.53–0.69)	<0.001
**Urinary tract infections**	444 (17.1)	317 (12.2)	1.48 (1.26–1.73)	1.59 (1.35–1.87)	<0.001
**Vasopressor use**	232 (8.9)	84 (3.2)	2.93 (2.27–3.79)	3.07 (2.35–3.99)	<0.001
**Mechanical ventilation**	976 (37.7)	349 (13.5)	3.88 (3.38–4.45)	5.23 (4.46–6.14)	<0.001
**Cardiogenic shock**	204 (7.9)	125 (4.8)	1.68 (1.34–2.12)	1.69 (1.32–2.15)	<0.001
**Septic shock**	394 (15.2)	165 (6.4)	2.63 (2.17–3.19)	2.86 (2.34–3.49)	<0.001
**Ventricular tachycardia**	0	0	-	-	-
**Outcomes**	**Diabetes Insipidus** **(n = 2590)**	**Without Diabetes** **Insipidus** **(n = 2590)**	**Coefficient (95% Confidence Interval)**		***p*-Value**
			**Unadjusted**	**Adjusted**	
**Cost**—median (IQR)	25,299 (11,596–53,420)	12,898 (6695–26,103)	0.60 (0.59–0.61)	0.55 (0.54–0.56)	<0.001
**Length of stay**—median (IQR)	7 (4–16)	5 (3–10)	0.43 (0.42–0.45)	0.44 (0.42–0.45)	<0.001

Represented as frequency (percentage) or median (interquartile range).

**Table 3:** Association between diabetes insipidus and 30-day readmissions in heart failure patients using multivariate regression model after propensity score matching.

Outcomes—n(%)	Diabetes Insipidus (n = 2590)	Without Diabetes Insipidus (n = 2590)	Odds Ratio (95% Confidence Interval)		*p*-Value
			Unadjusted	Adjusted	
**All-cause readmissions**	**358 (13.8)**	**393 (15.2)**	0.89 (0.76–1.04)	0.89 (0.76–1.04)	0.14
**Cardiac readmissions**	265 (74.1)	333 (84.7)	0.51 (0.35–0.73)	0.45 (0.30–0.67)	<0.001
**Non-cardiac readmissions**	93 (25.9)	60 (15.3)	1.94 (1.35–2.79)	2.21 (1.48–3.29)	<0.001
**Heart failure readmissions**	216 (8.4)	293 (11.3)	0.51 (0.38–0.70)	0.47 (0.33–0.66)	<0.001
**Composite of in-hospital mortality or 30-day all-cause readmissions**	1041 (40.2)	569 (21.9)	2.38 (2.11–2.69)	2.48 (2.19–2.81)	<0.001

Represented as frequency (percentage).

## Data Availability

The original contributions presented in the study are included in the article, further inquiries can be directed to the corresponding authors.

## Supplementary Materials

The following supporting information can be downloaded at: https://www.mdpi.com/article/10.3390/jcm14072308/s1.

## Abbreviations

AKI	Acute kidney injury
AVP	Arginine vasopressin
CCSR	Clinical Classifications Software Refined
CI	Confidence interval
ICD-10	International Classification of Diseases, Tenth Revision
ICU	Intensive Care Unit
MI	Myocardial infarction
NRD	National Readmission Database
NYHA	New York Heart Association
OR	Odds ratio
PCI	Percutaneous coronary intervention
PSM	Propensity score matching
SGLT2	Sodium-glucose cotransporter 2
UTI	Urinary tract infection
V1a and V2	Vasopressin receptors 1a and 2
