# Supplementary material for: Prognostic Implications of Diabetes Insipidus in Heart Failure Hospitalizations: Insights from the U.S. National Readmissions Database 2016–2021
Source: J Clin Med. 2025 Mar 28;14(7):2308. doi: 10.3390/jcm14072308 (PMC11989343; doi:10.3390/jcm14072308)
Supplement: Supplementary file 1 [file jcm-14-02308-s001.zip › jcm-3513964-supplementary.pdf]

SUPPLEMENTARY MATERIAL

Prognostic Implications of Diabetes Insipidus in Heart Failure Hospitalizations: Insights from a National Database 2016-2021

Lakshmi Menon[1], Shubhadarshini Pawar[2], Dileep Kumar Reddy Regalla[3]

Supplement Table S1: **International Classification of Diseases, Tenth Revision, Clinical Modification and Procedure Coding System (ICD-10-CM/PCS) codes used to identify baseline comorbidities, and procedures**

Variable	***ICD-10-CM/PCS***
Diabetes Insipidus	E232, N251
Heart failure	I50.1, I50.2x, I50.3x, I50.4x, I50.81x, I50.82, I50.83, I50.84, I50.89, I50.9
End stage renal disease	N186
Diabetes mellitus	E10, E11, E12, E13, E14
Smoking	Z72.0, Z87.891, F17.200, O99.33x
Dyslipidemia	E78.00, E78.01, E78.1, E78.2, E78.3, E78.41, E78.49, E78.5
Hypertension	Elixhauser Comorbidity Software
Obesity	Elixhauser Comorbidity Software
Known CAD	I25.xxx (except I25.2)
Prior MI	I25.2
Prior PCI	Z95.5, Z98.61
Prior coronary artery bypass grafting	Z95.1
Prior PPM/ICD	Z95.0, Z95.810
Prior TIA/Stroke	Z86.73
Peripheral artery disease	Elixhauser Comorbidity Software
Carotid artery disease	I65.21, I65.22, I65.23, I65.29
Chronic kidney disease	Elixhauser Comorbidity Software
Chronic pulmonary disease	Elixhauser Comorbidity Software
Chronic liver disease	Elixhauser Comorbidity Software
Cancer	Elixhauser Comorbidity Software
**Outcomes**	
Vasopressor use	3E030XZ, 3E033XZ, 3E040XZ, 3E043XZ, 3E050XZ, 3E053XZ, 3E060XZ, 3E063XZ
Acute kidney injury	N170, N171, N172, N178, N179
Hyponatremia	E871
Hypernatremia	E870
Acute Pulmonary Edema	J810
Chronic Pulmonary Edema	J811

Cerebral Edema	G936
Urinary tract infections	N300, N390
Mechanical ventilation	0BH17EZ, 0BH18EZ , 5A1935Z , 5A1945Z
Cardiogenic Shock	R570
Ventricular Tachycardia	I4720
Septic Shock	R6521
**Readmissions**	
Cardiac causes	The Clinical Classifications Software Refined (CCSR) for ICD-10-CM Diagnoses
Heart failure causes	The Clinical Classifications Software Refined (CCSR) for ICD-10-CM Diagnoses

Abbreviations: CABG, coronary artery bypass graft; MI, myocardial infarction; PCI, percutaneous coronary intervention;

Hypertension (complicated and uncomplicated), diabetes mellitus (complicated and uncomplicated), obesity, peripheral artery disease, chronic kidney disease, chronic liver disease, anemia, coagulopathy, dementia, hypothyroidism, pulmonary circulate; on disorders, and cancer were identified from the Elixhauser Comorbidity Software included in the Nationwide Readmissions Database.
